# Comparing the effects of propofol and sevoflurane anesthesia on cognitive dysfunction in elderly patients after abdominal surgery: a systematic review and meta-analysis of randomised controlled trials

**DOI:** 10.3389/fphar.2026.1807219

**Published:** 2026-06-17

**Authors:** Nana Guo, Xuxin Wen, Xiao Wang, Yonghua Wang, Yun Su, Tingxin Zhang

**Affiliations:** 1 Critical Care Medicine, Ordos Central Hospital, Ordos, China; 2 Department of Trauma and Sports Medicine, Ordos Central Hospital, Ordos, China; 3 Department of Orthopedics, Ordos Central Hospital, Ordos, China

**Keywords:** elderly patients, meta-analysis, postoperative cognitive dysfunction, propofol, sevoflurane

## Abstract

**Objectives:**

To conduct a systematic review and comparison of the effects of propofol and sevoflurane anesthesia on cognitive dysfunction in elderly patients following abdominal surgery.

**Methods:**

Comparative studies published in PubMed, Web of Science, EBSCOhost, Cochrane Library, EMBASE, and Medline databases as of 08 Jun 2025 were searched. The final extracted data was analyzed using Review Manager 5.4.

**Results:**

The 7 randomized controlled studies included a total of 802 patients who had undergone abdominal surgery, of whom 402 were anesthetized with propofol and 400 were anesthetized with sevoflurane. The outcomes of the meta-analysis showed that there were no statistically significant differences in operating time, anesthesia time, incidence of hypotension, the incidence of postoperative cognitive dysfunction (all P > 0.05), between the propofol and sevoflurane groups. Moreover, there was no significant difference in the severity of cognitive dysfunction between the two groups of patients on the 1st, 3rd, and 7th day after surgery (all P > 0.05).

**Conclusion:**

The meta-analysis results showed that there was no significant difference in the severity of cognitive dysfunction between the he propofol and sevoflurane groups of patients on the 1st, 3rd, and 7th day after surgery. This conclusion still does not prove that either one is more suitable for anesthesia in elderly patients, and more samples and clinical studies are needed to confirm.

**Trial Registration:**

PROSPERO registration number is: CRD42022324055.

## 1 Background

With the development of medical technology and the aggravation of the aging population, the rate of elderly patients undergoing surgical treatment is gradually increasing (Yin et al., 2024). At the same time, postoperative complications in elderly patients have also attracted more attention. Postoperative cognitive dysfunction (POCD) is one of the common postoperative complications in elderly patients, with a high incidence and a serious impact on the prognosis of patients (Steinmetz et al., 2009; Desai et al., 2022). POCD is associated with a variety of factors, including age (Monk et al., 2008; Feng et al., 2023), education level, genetic factors, surgery, anesthesia and so on (Janssen et al., 2019).

At present, many studies have confirmed that surgery and anesthesia can cause POCD. One study found that the incidence of POCD in elderly patients undergoing minor surgery was 6.8% at day 7% and 6.6% at 3 months (Canet et al., 2003). The occurrence of POCD is also related to different anesthesia methods. A meta-analysis of Sun et al. compared the effects of propofol and sevoflurane on postoperative cognitive function in elderly patients with lung cancer. The results showed that the adverse effects of propofol on postoperative cognitive function of elderly lung cancer patients were significantly greater than sevoflurane (Sun et al., 2019; Li et al., 2024). Another meta-analysis compared the effects of inhalation anesthesia and propofol anesthesia on postoperative cognitive dysfunction in elderly non-cardiac surgery patients. The study showed that the incidence of POCD based on propofol anesthesia was significantly lower than that of inhalation anesthesia 2–6 days after surgery, and the Mini-Mental State Examination (MMSE) scores based on propofol anesthesia were significantly higher than those after inhalation anesthesia (Pang et al., 2021).

So far, there are many methods to evaluate postoperative cognitive function, such as: the Mini-Mental State Examination (MMSE) score, Wechsler Adult Intelligence Scale (WAIS), Montreal Cognitive Assessment (MoCA), Loewenstein Occupational Therapy Cognitive Assessment (LOTCA) and so on. But the most widely used is the MMSE Score. Because it has the characteristics of high sensitivity, strong specificity, easy operation and less time consuming. The full score of MMSE is 30, and the higher the score, the less cognitive impairment (Folstein et al., 1975).

Studies have compared the effects of different anesthesia methods on postoperative cognitive dysfunction in elderly patients with lung cancer or patients undergoing non-cardiac surgery (Wang et al., 2023; Cao et al., 2023). However, the conclusions about the effects of propofol and sevoflurane on postoperative cognitive function of elderly patients undergoing abdominal surgery are still not uniform. The purpose of this meta-analysis was to explore which anesthesia method had less effect on postoperative cognitive function in elderly patients undergoing abdominal surgery.

## 2 Materials and methods

### 2.1 Search strategies

We searched the following databases, including: PubMed, Web of Science, EBSCOhost, Cochrane Library, EMBASE and Medline. A combination of Medical Subject Headings (MeSH) and keywords was used for retrieval, and the retrieval time was from the database construction time to 08 Jun 2025. We didt restrict searches based on language or year of publication. We conducted a manual search of references to published meta-analyses and review articles to avoid missing relevant studies.

### 2.2 Study selection

The two groups screened articles according to their titles and abstracts, and excluded articles that did not meet the inclusion criteria. If it was difficult to decide based on the abstract, the full text was retrieved. When the two groups had different opinions, consensus was reached through discussion, otherwise, differences were resolved through the third group.

### 2.3 Inclusion and exclusion criteria

Inclusion criteria: (1). Included studies were randomized controlled studies (RCTs); (2). Patients aged≥60 years with abdominal surgery; (3). Anesthesia with propofol or sevoflurane; (4). Outcomes included at least the incidence of postoperative cognitive dysfunction, MMSE score, operating time, anesthesia time or incidence of hypotension.

Exclusion criteria: (1). Editorials, reviews, letters, abstracts, animal experiments or conference proceedings; (2). The patient did not undergo abdominal surgery; (3). No relevant intervention or outcome.

### 2.4 Data extraction

A standardized data extraction table was made by us. This table contains basic study information for each eligible article, including: author, year of publication, title, country, study design, sample size, demographics, anesthesia methods, and comparison outcomes. The primary outcomes of this study were the MMSE score and the incidence of POCD. Secondary outcomes were operating time, anesthesia time, and incidence of hypotension. The data were separately extracted and compared by two groups of authors, quality assessment and verification procedures were performed afterwards. When the two groups had different opinions, consensus was reached through discussion, otherwise, differences were resolved through the third group.

### 2.5 Quality assessment

**FIGURE 1:**
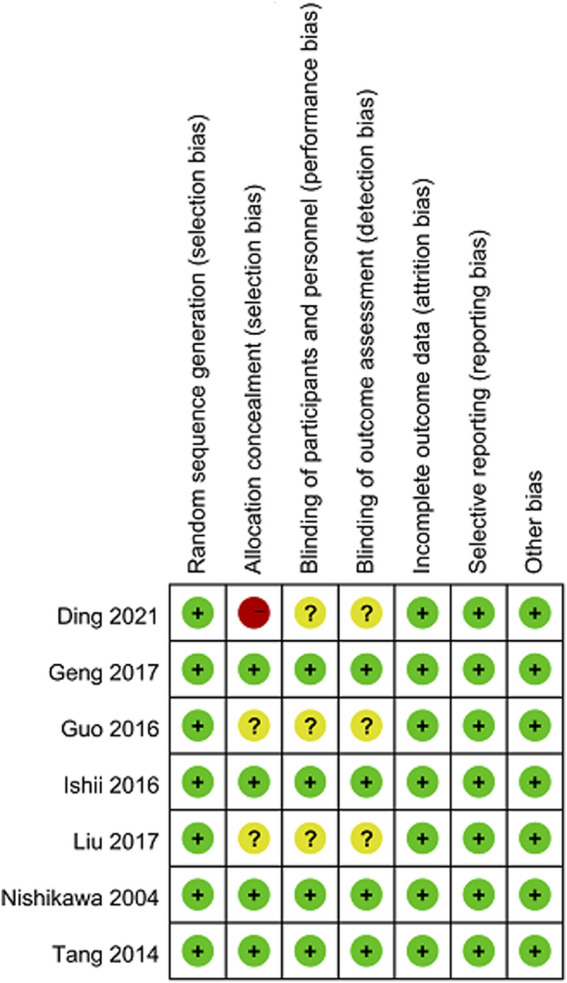
The methodological quality of the randomized controlled trials.

The Cochrane Handbook for Systematic Reviews of Interventions (Higgins et al., 2011) was used by us for quality assessment of RCTs. These include the following: random sequence generation, allocation concealment, blinding of participants and personnel, blinding of outcome assessment, incomplete outcome data, selective outcome reporting, and other sources of bias (Figure 1). Quality assessment will be carried out by two authors separately, and any objections will be discussed with a third party.

### 2.6 Statistical analyses

Review Manager Version 5.4 (Copenhagen: The Nordic Cochrane Centre, The Cochrane Collaboration) was used to analyse the data. For continuous variables, such as operating time, anesthesia time, and the MMSE Score, the means and standard deviations were pooled to a weighted mean difference (WMD) and 95% confidence interval (CI). Odds ratios (ORs) and 95% CIs were used to evaluate dichotomous variables, such as the Incidence of POCD. I^2^ was used to quantify heterogeneity. I^2^>50% indicated significant heterogeneity, and a random effects model was used to estimate the unstandardized mean difference. Otherwise, a fixed-effects model was applied.

## 3 Results

### 3.1 Study selection

**FIGURE 2:**
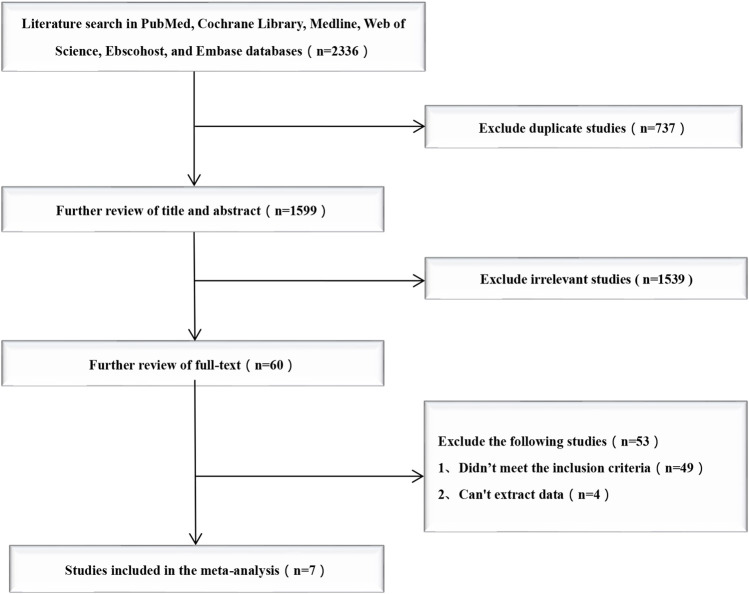
The article screening flow chart.

Initially, 2336 studies were retrieved from the databases, leaving 1599 after duplicates were removed. After removing irrelevant studies by title and abstract, 60 studies remained. By reading the full text, we found that 49 studies did not meet the inclusion criteria, and 4 studies did not report the results of this study. Ultimately, we included 7 studies (Tang et al., 2014; Guo et al., 2016; Ishii et al., 2016; Nishikawa et al., 2004; Geng et al., 2017; Liu et al., 2017; Ding et al., 2021) and analyzed the extracted data. The article screening flow chart is shown in Figure 2.

### 3.2 Study characteristics

The 7 studies included a total of 802 patients who had undergone abdominal surgery, of whom 402 were anesthetized with propofol and 400 were anesthetized with sevoflurane. Clinical data such as age, gender, body weight, body mass index (BMI), American Society of Anesthesiologists (ASA) grade, and type of operation were collected for each study. After data analysis, the results showed that there were no significant differences in these indicators, proving the comparability (p > 0.05) (Table 1).

**TABLE 1:** Characteristics of included studies.

Author (year)	Country	Sex (M/F)		Age (years)		Type of surgery	ASA grade	Outcomes
		Propofol group	Sevoflurane group	Propofol group	Sevoflurane group			
Tang (2014)	China	26/75	32/67	69.6 ± 4.8	70.0 ± 4.3	Radical rectal resection surgery (miles type)	I–III	1,2, 5–7
Guo (2016)	China	23/9	23/8	63.5 ± 7.6	64.2 ± 8.3	Elective abdominal surgery	I–III	3, 5, 6, 7
Ishii (2016)	Japan	20/9	20/10	77.3 ± 4.6	76.5 ± 4.5	Elective gastrectomy, colectomy, or rectectomy	I–II	1, 6, 7
Nishikawa (2004)	Japan	13/12	12/13	71 ± 8	71 ± 7	Elective laparoscope-assisted surgical procedures	I–II	1,2, 6, 7
Geng (2017)	China	20/30	22/28	≥65	≥65	Laparoscopic cholecystectomy	II–III	1, 2, 3, 4
Liu (2017)	China	31/25	31/25	74.16 ± 4.21	75.82 ± 4.17	Laparoscopic colorectal resection	-	1, 3, 4,7
Ding (2021)	China	35/30	34/31	74.8 ± 6.21	75.1 ± 6.45	Abdominal surgery: Rectal surgery colon surgery	I-II	3–7

ASA: american society of anesthesiology; Outcomes: 1. The incidence of POCD; 2. The incidence of hypotension; 3. MMSE, score at 1 day after surgery; 4. MMSE, score at 3 days after surgery; 5. MMSE, score at 7 days after surgery; 6. Operating time; 7. Anesthesia time.

### 3.3 The incidence of POCD

**FIGURE 3:**
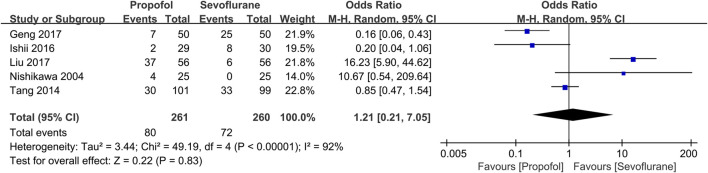
Meta-analysis of patients receiving propofol versus sevoflurane anesthesia for the incidence of POCD in patients receiving propofol or sevoflurane anesthesia.

A total of five studies (Tang et al., 2014; Ishii et al., 2016; Nishikawa et al., 2004; Geng et al., 2017; Liu et al., 2017) were included, including 261 patients in the propofol group (group P) and 260 in the sevoflurane group (group S). Meta-analysis showed no significant difference in the incidence of POCD between the two groups (OR, 1.21; 95% CI, 0.21 to 7.05; P > 0.05). Heterogeneity test result was I^2^ = 92%, and the random effects model was used (Figure 3).

### 3.4 MMSE score after surgery

#### 3.4.1 MMSE score at 1 day after surgery

**FIGURE 4:**
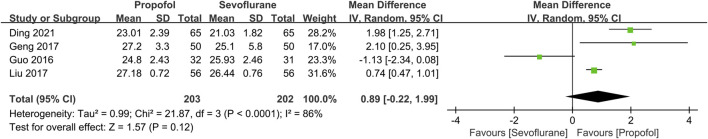
Meta-analysis of patients receiving propofol versus sevoflurane anesthesia for the MMSE score at 1 day after surgery.

Four studies (Guo et al., 2016; Geng et al., 2017; Liu et al., 2017; Ding et al., 2021) compared MMSE score on day 1 after surgery in 203 patients in the group P and 202 patients in the group S. Meta-analysis results showed that there was no significant difference in MMSE score between the two groups, but MMSE score in group P was slightly higher than that in group S (WMD, 0.89; 95% CI, −0.22 to 1.99; P > 0.05). Heterogeneity test results (I^2^ = 86%) showed significant heterogeneity (Figure 4).

#### 3.4.2 MMSE score at 3 days after surgery

**FIGURE 5:**
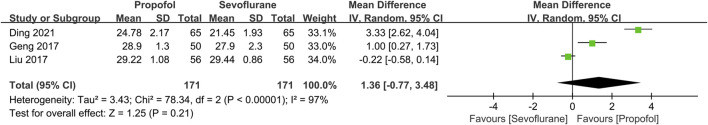
Meta-analysis of patients receiving propofol versus sevoflurane anesthesia for the MMSE score at 3 days after surgery.

A total of three studies (Geng et al., 2017; Liu et al., 2017; Ding et al., 2021) were included, with 171 patients in the group P and 171 patients in the group S. Similar to the MMSE score results on the 1st postoperative day, the MMSE scores of the two groups on the 3rd postoperative day were not statistically significant, but the MMSE score of the group P was still slightly higher than that of the group S (WMD, 1.36; 95% CI, −0.77 to 3.48; P > 0.05). The result of the heterogeneity test was I^2^ = 97%, indicating significant heterogeneity (Figure 5).

#### 3.4.3 MMSE score at 7 days after surgery

**FIGURE 6:**
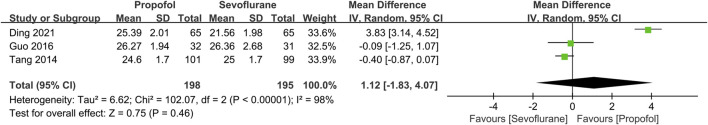
Meta-analysis of patients receiving propofol versus sevoflurane anesthesia for the MMSE score at 7 days after surgery.

Three studies (Tang et al., 2014; Guo et al., 2016; Ding et al., 2021) assessed MMSE score on day 7 after surgery. 198 patients were enrolled in the group P and 195 patients in the group S. The Meta-analysis results showed that the MMSE scores of the two groups were not statistically significant (WMD, 1.12; 95% CI, −1.83 to 4.07; P > 0.05). The result of the heterogeneity test was I^2^ = 98%, indicating significant heterogeneity (Figure 6).

### 3.5 Operating time

**FIGURE 7:**
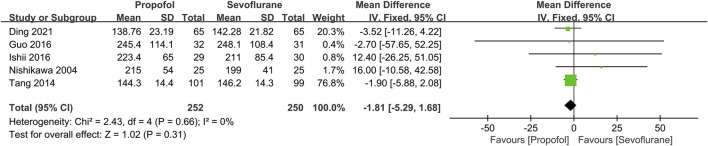
Meta-analysis of patients receiving propofol versus sevoflurane anesthesia for the operating time.

Five studies (Tang et al., 2014; Guo et al., 2016; Ishii et al., 2016; Nishikawa et al., 2004; Ding et al., 2021) compared operative time. The number of patients in the two groups were 252 (the group P) and 250 (the group S). The results showed that P = 0.66, indicating that there was no significant difference in operation time between the two groups (WMD, −1.81; 95% CI, −5.29 to 1.68; P > 0.05). Heterogeneity test result was I^2^ = 0%, and the fixed effects model was used (Figure 7).

### 3.6 Anesthesia time

**FIGURE 8:**
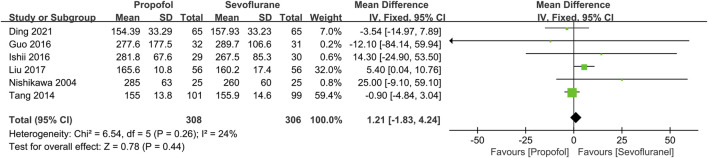
Meta-analysis of patients receiving propofol versus sevoflurane anesthesia for the anesthesia time.

A total of six studies (Tang et al., 2014; Guo et al., 2016; Ishii et al., 2016; Nishikawa et al., 2004; Liu et al., 2017; Ding et al., 2021) compared the duration of anesthesia. The number of patients enrolled was 308 in the group P and 306 in the group S. The results showed that there was no significant difference in anesthesia time between the two groups (P = 0.44) (WMD, 1.21; 95% CI, −1.83 to 4.24; P > 0.05). Heterogeneity test result was I^2^ = 24%, and the fixed effects model was used (Figure 8).

### 3.7 Incidence of hypotension

**FIGURE 9:**
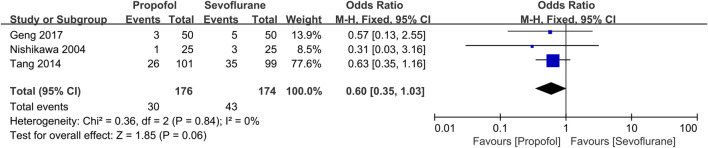
Meta-analysis of patients receiving propofol versus sevoflurane anesthesia for the incidence of hypotension.

Three studies (Tang et al., 2014; Nishikawa et al., 2004; Geng et al., 2017) compared the incidence of intraoperative hypotension. 176 patients were enrolled in the group P and 174 in the group S. Meta-analysis results showed that the incidence of intraoperative hypotension in the two groups was not statistically significant (P = 0.06), but the incidence of hypotension in the group P was slightly lower than that in the group S (RR: 0.60; 95% CI, 0.35 to 1.03; P > 0.05). Heterogeneity test result was I^2^ = 0%, and the fixed effects model was used (Figure 9).

### 3.8 Sensitivity analysis

Sensitivity analysis was performed using the leave-one-out method to assess the robustness of pooled estimates. For the incidence of POCD, after omitting each study individually, the pooled OR ranged from 0.65 to 1.84 (all 95% CI included 1, all P > 0.05). The overall conclusion of no significant difference between propofol and sevoflurane remained unchanged. For MMSE score at postoperative day 1,after sequential exclusion of each study, the pooled WMD ranged from 0.42 to 1.31 (all 95% CI included 0, all P > 0.05). The result remained stable and non-significant. For MMSE score at postoperative day 3,after leave-one-out exclusion, the pooled WMD ranged from 0.78 to 1.72 (all 95% CI included 0, all P > 0.05). The conclusion was consistent and robust. For MMSE score at postoperative day 7, after excluding each study individually, the pooled WMD ranged from 0.69 to 1.45 (all 95% CI included 0, all P > 0.05). No change to the primary conclusion was observed. All sensitivity tests confirmed that the pooled results were stable and reliable, and the finding of no significant difference in postoperative cognitive function between propofol and sevoflurane was consistent and not driven by any single study.

## 4 Discussion

The results of the above meta-analysis showed that when there was no significant difference in the operation time and anesthesia time, although the postoperative MMSE score of the two groups was not statistically significant, the MMSE score of the group P was slightly higher than that of the group S, and the difference in MMSE score between the two groups was significantly reduced on the 7th day after surgery.

POCD typically occurs within 7 days after surgery or before discharge and is characterized by temporary changes in mental status, concentration, and level of consciousness (Meyburg et al., 2017). It commonly arises in the operating room or postoperative recovery room, during general anesthesia, or shortly after the surgical procedure (Chen et al., 2022). The specific pathophysiological mechanism of POCD is currently unclear. It is widely believed that a combination of predisposing and precipitating factors contribute to the development of POCD. Early identification of risk factors is considered an effective approach to reducing the risk of POCD (Liu J. et al., 2022 ). These risk factors include advanced age, comorbidities, preoperative fasting, the type of surgery (abdominal and cardiothoracic), intraoperative bleeding, prolonged surgery time, intraoperative electrolyte imbalance, and postoperative pain (Thedim and Vacas, 2024; Tian et al., 2017). Potential causes of POCD may involve oxidative stress, brain structure or function damage, neurotransmitter imbalances, and disturbances in thermoregulation. At present, the specific pathogenesis of POCD induced by general anesthetics has not been fully elucidated. Brain-gut axis may be one of the pathogenesis (Zhu et al., 2025; Wang et al., 2025). The brain-gut axis is a bidirectional communication system between the brain and gut microbes, which mainly involves the afferent and efferent signals of various aspects such as nerve, endocrine, immune and metabolic pathways (Dinan and Cryan, 2017). Guo et al. conducted continuous intravenous infusion of propofol intervention in wistar rats. Feces of each rat were collected before intervention and at day 1, 3, 7 and 14 after intervention, and 16SRNA sequencing analysis was performed. The results showed that continuous intravenous infusion of propofol had little effect on intestinal flora of rats (Guo et al., 2021). Han et al. verified the effects of sevoflurane inhalation anesthesia on the intestinal microbiome of mice, and the results showed that there were significant differences in species abundance between the experimental group and the control group on the first, third and seventh days after anesthesia. On day 14, although there was still a significant difference between the two groups, the degree of difference was lower than the previous several time points. There were also significant differences in species composition between the two groups (Han et al., 2021). For patients undergoing abdominal surgery, the dual effects of the primary disease and anesthetic drugs may further alter intestinal flora status, which may indirectly affect postoperative cognitive function through the brain-gut axis, but this potential correlation requires validation by more clinical studies with direct evidence.

On the other hand, propofol and sevoflurane are commonly used in clinical general anesthesia, mostly used in elderly patients, but they have different mechanisms of anesthesia. Propofol inhibits spinal cord neurons by GABAA receptor mediated only, while the inhibitory effect of sevoflurane on spinal cord neurons is mediated by glycine receptors and GABAA receptors (Grasshoff and Antkowiak, 2004). GABAA receptors and glycine receptors are considered to be closely related to cognitive functions such as learning and memory and the formation of reflex activities in the central nervous system (Kozinn et al., 2006). Other studies have shown that after sevoflurane inhalation, the biological function of acetylcholine receptors in the postsynaptic membrane is inhibited, thus reducing the transmission of central excitatory neurotransmitters. On the other hand, the mitochondrial membrane potential of nerve cells under the direct action of sevoflurane induces the release of cytochrome C, leading to apoptosis. All the above mechanisms will eventually affect the cognitive function of patients [18]. However, the anesthetic effect of propofol is mediated by the release of neurotransmitters in the presynaptic membrane, and its inhibitory effect on the central nervous system is weak, so its effect on postoperative cognitive function of patients is also small (Liu B. et al., 2022). It can be seen that propofol and sevoflurane achieve the anesthetic effect through different mechanisms. Propofol has relatively little effect on the central nervous system, so its influence on the postoperative cognitive function of patients is relatively small. Our meta-analysis results showed that although the postoperative MMSE score of the two groups was not statistically significant, the MMSE score of group P was slightly higher than that of group S, which still needs to be confirmed by more samples and clinical studies.

Notably, our meta-analysis showed extremely high heterogeneity (I^2^ = 92% for POCD incidence; I^2^ = 86%–98% for MMSE scores). The wide confidence intervals (e.g., OR = 1.21, 95% CI: 0.21–7.05) indicate considerable uncertainty in the pooled effect size, which should be interpreted with extreme caution to avoid misleading clinical decision-making. To validate the reliability of our findings despite high heterogeneity, we performed a leave-one-out sensitivity analysis. The results confirmed that the overall conclusion of no significant difference between propofol and sevoflurane was robust and not affected by any single included study. Formal subgroup analysis was not performed because the limited number of RCTs (n = 7) and lack of detailed stratified data in original studies would lead to insufficient statistical power and unreliable results.

This is the first meta-analysis to compare the effects of propofol and sevoflurane anesthesia on postoperative cognitive dysfunction in elderly patients undergoing abdominal surgery, but the study has several notable limitations. First, only 7 randomized controlled trials with a total of 802 patients were included, which limits the statistical power of the pooled analyses. Second, formal subgroup analysis was not feasible due to the small number of included studies and insufficient stratified data; however, sensitivity analysis was performed and verified the stability of the main conclusion. Extremely high heterogeneity was observed across studies, which reduced the precision of pooled estimates and resulted in wide confidence intervals. Third, there is currently no universally accepted unified diagnostic standard for POCD, and MMSE score assessment may be influenced by patients’ educational level and the subjective judgment of assessors. Fourth, the follow-up duration was limited to 7 days after surgery. Long-term cognitive outcomes at 3 months, 6 months, or longer were not evaluated. Therefore, our findings only reflect short-term cognitive changes and cannot be used to infer long-term cognitive effects.

To address the heterogeneity and limitations identified in this study, and to provide more reliable clinical evidence for anesthesia selection in elderly patients undergoing abdominal surgery, future randomized controlled trials should prioritize several key aspects: unifying the diagnostic criteria for POCD and standardizing the implementation of cognitive assessment tools such as the MMSE; standardizing the study population by focusing on a single type of abdominal surgery and collecting detailed baseline clinical data to ensure homogeneity; unifying anesthetic and perioperative management protocols, including anesthetic dosage, adjuvant drug use, intraoperative hemodynamic management, and postoperative analgesia, to ensure intervention consistency; and conducting large-sample, multi-center studies to improve statistical power and the generalizability of study results.

## 5 Conclusion

The results of this meta-analysis showed that there was no statistically significant differences in the incidence of postoperative cognitive dysfunction between the propofol and sevoflurane groups. Moreover, there was no significant difference in the severity of cognitive dysfunction between the two groups of patients on the 1st, 3rd, and 7th day after surgery. Current evidence cannot prove that propofol is more suitable for anesthesia in elderly patients than sevoflurane, and more samples and clinical studies are needed in the future to confirm.
